# Effects of concomitant inactivation of p53 and pRb on response to doxorubicin treatment in breast cancer cell lines

**DOI:** 10.1038/cddiscovery.2017.26

**Published:** 2017-05-22

**Authors:** Johanna Huun, Per Eystein Lønning, Stian Knappskog

**Affiliations:** 1 Section of Oncology, Department of Clinical Science, University of Bergen, Bergen, Norway; 2 Department of Oncology, Haukeland University Hospital, Bergen, Norway

## Abstract

Loss of *TP53* and *RB1* function have both been linked to poor response to DNA damaging drugs in breast cancer patients. We inactivated *TP53* and/or *RB1* by siRNA mediated knockdown in breast cancer cell lines varying with respect to ER/PgR and Her-2 status as well as *TP53* and *RB1* mutation status (MCF-7, T47D, HTB-122 and CRL2324) and determined effects on cell cycle arrest, apoptosis and senescence with or without concomitant treatment with doxorubicin. In T47D cells, we found the cell cycle phase distribution to be altered when inactivating *TP53* (*P*=0.0003) or *TP53* and *RB1* concomitantly (*P*≤0.001). No similar changes were observed in MCF-7, HTB-122 or CRL2324 cells. While no significant change was observed for the CRL2324 cells upon doxorubicin treatment, MCF-7, T47D as well as HTB-122 cells responded to knockdown of *TP53* and *RB1* in concert, with a decrease in the fraction of cells in G1/G0-phase (*P*=0.042, 0.021 and 0.027, respectively). Inactivation of *TP53* and/or *RB1* caused no change in induction of apoptosis. Upon doxorubicin treatment, inactivation of *TP53* or *RB1* separately caused no induction of apoptosis in MCF-7 and HTB-122 cells; however, concomitant inactivation leads to a slightly reduced activation of apoptosis. Interestingly, upon doxorubicin treatment, concomitant inactivation of *TP53* and *RB1* caused a decrease in senescence in MCF-7 cells (*P*=0.027). Comparing the effects of concomitant knockdown on apoptosis and senescence, we observed a strong interaction (*P*=0.001). We found concomitant inactivation of *TP53* and *RB1* to affect various routes of response to doxorubicin treatment in breast cancer cells.

## Introduction

Chemotherapy resistance is the main cause of therapy failure and death among cancer patients; yet the mechanisms behind resistance remains poorly understood.^1^

Doxorubicin causes DNA double helical breaks leading to activation of cell cycle arrest, apoptosis or entry of a permanent state of cell cycle arrest, senescence.^2–4^

In regulation of these processes, the tumor suppressor’s p53 (encoded by the *TP53* gene) and the retinoblastoma protein, pRb (encoded by the *RB1* gene) are both known to have key roles.^5–7^

*TP53* mutations have been associated with resistance to DNA damaging chemotherapy treatment across different malignancies.^8–12^ However, the finding that anthracyclines may work on some tumors harboring *TP53* mutations while other tumors respond to therapy despite lack of *TP53* function has lead us to postulate that redundant gene pathways may act in concert.^1,13^

pRb, regulating the transition between G1/G0- and S-phase in the cell cycle, has a key role executing cell cycle arrest in response to DNA damage.^14,15^ While conflicting evidence has linked *RB1* mutations to lack of, as well as an improved response to chemotherapy;^16,17^ previously we reported mutations in *RB1* to be associated with a poor response to anthracyclines and mitomycin in breast cancer.^18,19^

Experimental evidence has linked concomitant inactivation of the p53- and the Rb-pathway to lymphoma development and resistance toward cyclophosphamide in mice through prevention of cellular senescence,^20^ as well as immortalization of keratinocytes.^21^ While we found concomitant inactivation of the p53- and Rb-functional pathways to be associated with resistance to DNA damaging drugs in breast cancer patients, the mechanism behind this effect has not been elucidated.^22^

In order to further characterize the mechanisms behind chemotherapy resistance, we here performed functional analyzes of the cellular responses, cell cycle arrest, apoptosis and senescence upon siRNA mediated inactivation of *TP53* and *RB1*, alone or combined, in unstressed cells and after doxorubicin treatment in different breast cancer cell lines.

## Results

### Establishment of a concomitant knockdown model

**Figure 1:**
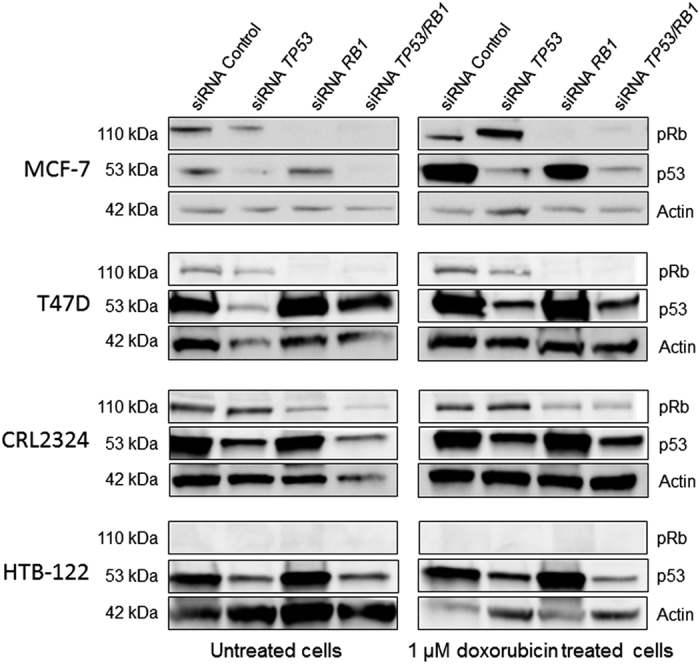
Protein expression after siRNA transfection of 4 cell lines. Left panel shows results of western blotting from untreated cells (DMSO), while the right panel show the corresponding results from cells treated with 1 *μ*M doxorubicin for 24 h. The lanes show results from cells subjected to siRNA-control and siRNA-mediated knockdown of *TP53*, *RB1* and these two genes concomitantly. The top rows presents pRb (110 kDa) and the middle lanes p53 (53 kDa). Actin is used at loading control (42 kDa).

In order to establish a model appropriate for studying the effects of lack of p53 and/or pRb on response to doxorubicin treatment, we applied siRNA-mediated knockdown. The siRNAs used effectively and selectively suppressed both *TP53* and *RB1* in untreated MCF-7 cells as well as in doxorubicin treated cells (Figure 1). Notably, *TP53* inactivation lead to a slight upregulation of pRb in chemotherapy treated cells, supporting the hypothesis that the Rb-pathway may act as compensatory pathway in *TP53* inactivated tumor cells in response to cellular stress. Efficient knockdown was also confirmed by western blot analysis in the additional cell lines T47D, HTB-122 and CRL2324 (with the expected exception that pRB could not be detected in HTB-122 cells, since these cells are known to harbor a homozygous frameshift deletion within the *RB1* gene).

Subsequently, we assessed the potential impact of knockdown of *TP53* or *RB1*, as well as both genes concomitantly, on three different cellular endpoints potentially induced by doxorubicin treatment: cell cycle arrest, apoptosis and senescence.

### Effect of *TP53* and/or *RB1* inactivation on cell cycle progression

**Figure 2:**
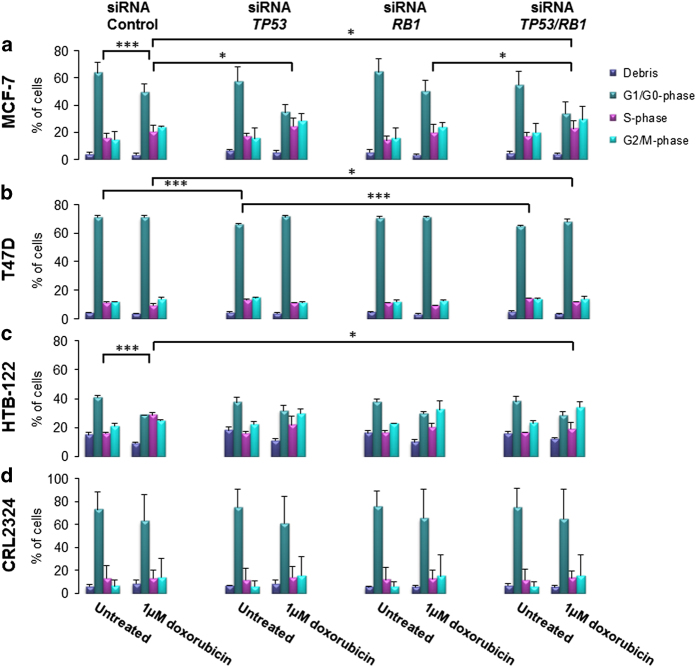
Effect on cell cycle progression. Cell cycle analysis after siRNA knockdown of untreated (DMSO) or 1 *μ*M doxorubicin treated (**a**) MCF-7, (**b**) T47D, (**c**) HTB-122 and (**d**) CRL2324 breast cancer cells. Cells were treated with siRNA-Control, siRNA against *TP53*, siRNA for *RB1* or concomitantly against *TP53* and *RB1*. Analysis was performed by NucleoCounter 3000. Bars present the percent of cell debris (purple), cells in G1/G0-phase (green), in S phase (pink) and in G2/M phase (blue), respectively. The experiment was repeated in triplicate, including three independent siRNA transfections and treatment with three different batches of DMSO or chemotherapy. Error bars indicate standard deviations. *P≤0.05, ***P≤0.001.

First we assessed the impact of *TP53* and *RB1* inactivation in MCF-7 cells not treated with any chemotherapy. Inactivation of *TP53*, *RB1* or both genes in concert, did not alter the cell cycle phase distribution significantly (Figure 2a). However, in a second ER-positive cell line, T47D, *TP53* knockdown lead to a significant difference in the cell cycle distribution compared to the siRNA-control (*P*≤0.001; Figure 2b). Knockdown of *RB1* caused no changes in cell cycle distribution in T47D cells, while concomitant inactivation of *TP53* and *RB1* lead to a more pronounced shift in the cell cycle compared to knockdown of *TP53* alone (analysis of variance with all phases included; *P*≤0.001). Performing the same experiments on triple negative cell lines (HTB-122 and CRL2324), we observed no direct effect with respect to cell cycle distribution upon knockdown of either *TP53* or *RB1* or by the two genes in concert (Figures 2c and d).

Doxorubicin treatment of the cell lines lead to a significant shift in the cell cycle phase distribution in MCF-7 cells (*P*=0.001; comparison of untreated and doxorubicin treated siRNA-control cells), while the T47D cells seemed not to respond to the dose applied and revealed no shift in the cell cycle. Regarding the triple negative cell lines, we found HTB-122 to have a significant shift in the cell cycle phase distribution upon doxorubicin treatment (variance analysis with all phases included*; P*≤0.001), while CRL2324 appeared to not respond to the treatment.

Assessing the impact of *TP53* inactivation on the response to doxorubicin treatment in MCF-7, we found the cell cycle phase distribution to alter significantly when compared with the siRNA-control cells (28% decrease in cells in the G1/G0-phase with a 20% increase of cells in the G2/M-phase: *P*=0.024; Figure 2a). A similar albeit non-significant trend was observed in HTB-122 cells (*P*=0.073); in contrast, no effects were recorded in T47D or CRL2324 cells (Figures 2b–d). *RB1* inactivation alone, on the other hand, had no effects on cell cycle distribution across the different cell lines.

Importantly, in doxorubicin treated MCF-7 cells, concomitant inactivation of *TP53* and *RB1* lead to decrease of cells arrested in G1/G0-phase and increase of cells in G2/M-phase compared to the siRNA-control cells (*P*=0.042), however, not significantly when compared to cells with *TP53* inactivated alone. Similar to MCF-7, in T47D cells, in which doxorubicin had revealed no effect on cell cycle in the siRNA-control cells, concomitant inactivation of *TP53* and *RB1* caused the same effect (*P*=0.021), indicating the cell cycle to be strongly affected by *TP53* and *RB1* inactivation under doxorubicin treatment. Among the triple negative cell lines, similar results were observed for HTB-122 (*P*=0.027), while no significant change was observed for the CRL2324 cells.

### Effect *TP53* and/or *RB1* inactivation on induction of apoptosis

Based on the changes observed in the cell cycle profiles, we further assessed the degree of initiated apoptosis after separate or concomitant inactivation of *TP53* and *RB1*.

**Figure 3:**
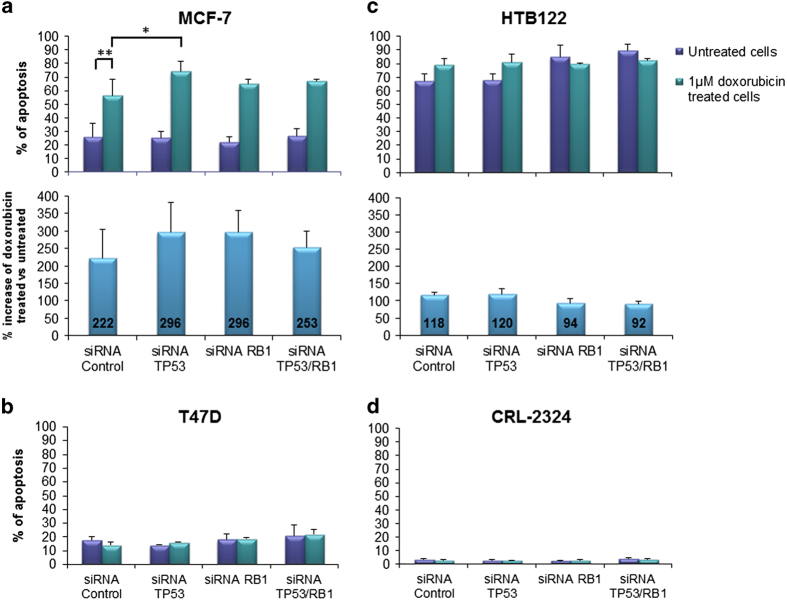
Induction of apoptosis by Annexin V analysis. (**a**) MCF-7, (**b**) T47D, (**c**) HTB-122 and (**d**) CRL2324 breast cancer cells were treated with siRNA-Control, siRNA for *TP53*, siRNA for *RB1* or siRNA against both *TP53* and *RB1* together. Graphs show the percentage of apoptotic cells; untreated (DMSO; purple bars) or treated with 1 *μ*M doxorubicin (green bars) for 24 h, analyzed by Annexin V assay 48 h post knockdown. The pillars represent both the cells that are early apoptotic and apoptotic. The experiment was repeated in triplicate, with three independent siRNA transfections and three different DMSO or doxorubicin batches. Error bars indicate standard deviations. *P≤0.05, **P≤0.01. The percentage change in untreated vs doxorubicin treated cells is presented for (**a**) MCF-7 and (**c**) HTB-122 (lower panel, blue bars).

In untreated cells, knockdown of *TP53*, *RB1* or both genes simultaneously did not reveal any major changes in the induction of apoptosis in either of the cell lines analyzed (Figures 3a–d).

Upon treatment with doxorubicin, the fraction of apoptotic MCF-7 cells increased significantly (*P*=0.002, comparison of siRNA-control treated cells; Figure 3a). Similar to our observations when assessing cell cycle progression, treatment with doxorubicin did not influence apoptosis in T47D and CRL2324 cells, while HTB-122 revealed a higher general level of apoptosis than MCF-7, but a more modest increase in apoptosis in response to doxorubicin (Figures 3b–d).

In cells treated with doxorubicin, inactivation of *TP53* or *RB1* alone or combined, caused an unexpected, slight increase in the fraction of apoptotic MCF-7 cells as compared to the siRNA-control. In contrast, no change was observed in HTB-122 cells (Figures 3a and c).

Interestingly, in MCF-7 cells, concomitant inactivation of *TP53* and *RB1* lead to a slightly reduced induction of apoptosis upon doxorubicin treatment as compared with when the two genes were inactivated separately (Figure 3a). A similar observation was made in the HTB-122 cells (Figure 3c). Taking the lack of reduced apoptosis (when comparing concomitant inactivation with siRNA-control) into account, these data indicate apoptosis not to be a cellular endpoint strongly affected by *TP53* and/or *RB1* inactivation.

### Effect of *TP53* and/or *RB1* inactivation on induction of senescence

In order to test a more long term effect of *TP53* and *RB1* inactivation and a possible alternative mechanism of response to doxorubicin, we assessed the induction of cellular senescence in MCF-7 cells with and without doxorubicin treatment.

**Figure 4:**
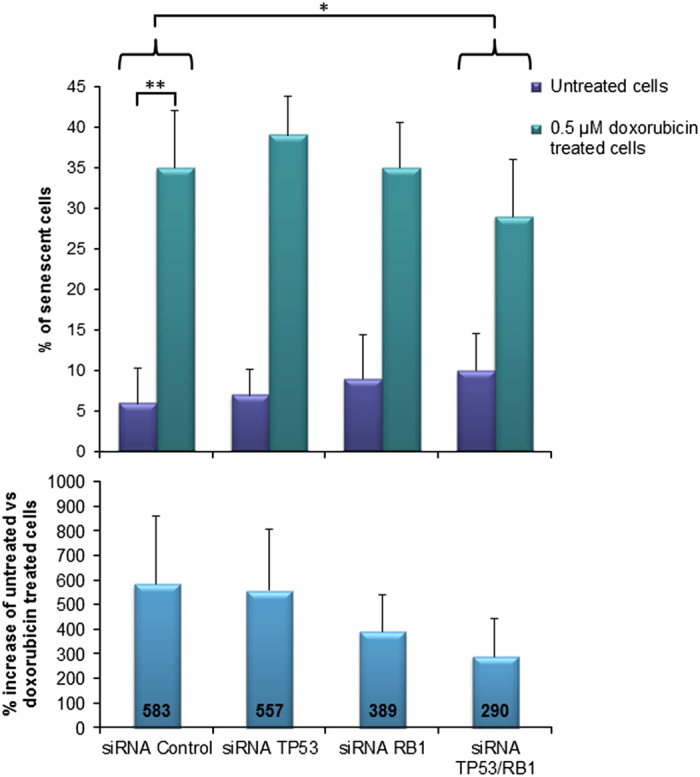
Induction of senescence measured by *β*-galactosidase activity staining. Bars indicate the percentage of senescent MCF-7 cells: untreated (0.5 *μ*M DMSO; purple bars) or cells treated with 0.5 *μ*M doxorubicin (green bars) for 7 days. Cells were treated with siRNA-Control, siRNA against *TP53*, *RB1* or both against *TP53* and *RB1* simultaneously. The percentage change in induced senescence of untreated vs doxorubicin treated cells is presented (lower panel, blue bars). Error bars indicate standard deviations. *P≤0.05, **P≤0.01.

In cells not exposed to doxorubicin, *TP53* and/or *RB1* inactivation (alone or combined) did not influence the fraction of cells undergoing senescence (Figure 4).

Upon treatment of siRNA-control cells with doxorubicin, we observed a large increase in the fraction of senescent cells (583% increase when compared to the untreated siRNA-control cells, *P*=0.004; Figure 4).

Most importantly, upon concomitant inactivation of *TP53* and *RB1*, doxorubicin treatment lead to a much smaller increase of senescent cells as compared to siRNA-control (290%; *P*=0.027 for interaction between concomitant knockdown and reduced induction of senescence).

Notably, we found a significant interaction when comparing the increase in apoptosis and the decrease in senescence in doxorubicin treated MCF-7 cells with concomitant inactivation of *TP53* and *RB1* (*P*=0.001).

## Discussion

The mechanism behind chemotherapy resistance is still poorly understood, despite extensive experimental research. While *TP53* and/or *RB*1 mutational status have been linked to lack of sensitivity to anthracyclines,^8,9,12,18,19,22^ the mechanisms remains poorly understood. Here, we aimed at systematically exploring the effect of *TP53* and *RB1* inactivation, separately and in concert, on the cellular response to doxorubicin treatment using a panel of cell lines representing various breast cancer subtypes (luminal, tipple negative and Her-2-classes), respectively.^23^ We used MCF-7 (ER+, PgR+ and Her-2 negative) being WT for *TP53* as well as for *RB1*, T47D (ER+, PgR+ and Her-2 negative) harboring mutated *TP53* (L194F) and WT *RB1*, HTB-122 (triple negative) harboring mutated *TP53* (R249S) and additionally a partly deleted *RB1*, likely causing a non-functional pRb protein and finally, CRL2324 (triple negative) also with mutated *TP53* (R175H) and WT *RB1*. For all three *TP53* mutated cell lines, the mutations are previously characterized as GOF mutations, and all three cell lines have lost their wild-type allele.

In our study, we applied siRNA-mediated knockdown of *TP53* and *RB1* in all the four cell lines, regardless of their mutational status. This was done partly in order to obtain a uniform comparison of the cell lines, however most importantly because it is unclear which (if any) normal p53 functions that are retained, and which additional functions that are gained by the different GOF mutations. siRNA mediated knockdown would therefore assure complete loss of p53 function, even in the cell lines harboring *TP53* mutations.

The effect and biological outcome of doxorubicin treatment *in vitro* is context dependent and the concentration of drug and time of exposure is crucial.^24,25^ The dosage of the doxorubicin treatment and the time of exposure used in the present study were chosen based on *in vivo* concentrations under treatment of breast cancer patients. Although this dose is somewhat lower than what is often used for *in vitro* studies, the finding of substantial effects of doxorubicin at this concentration on cell cycle progression in MCF-7 and HTB-122 cells revealed the drug concentration to be of biological importance.

In the present study we found inactivation of *TP53* and *RB1* in concert in general to have a larger effect on cell cycle distribution than inactivation of either of the two genes individually in MCF-7 cells. When addressing activation of apoptosis, however, upon concomitant inactivation of the two genes, we observed a somewhat unexpected increase in apoptotic cells; this may indicate that apoptosis might not be the most important cellular endpoint affected by *TP53* and/or *RB1* inactivation in MCF-7 cells. As an alternative endpoint of response to chemotherapy treatment, we also examined the induction of senescence in these cells. Interestingly, we observed a strong effect of concomitant inactivation of *TP53* and *RB1* on senescence. Notably, our finding of reduced senescence upon concomitant inactivation of *TP53* and *RB1* is in agreement with results from experimental studies revealing concomitant inactivation of the *TP53* and *RB1* pathways to be associated with lymphogenesis as well as resistance toward chemotherapy due to loss of senescence.^20^ More recently, Laine and colleagues have shown that in breast cancer cells with inactivated p53-p21 pathway, the feedback mechanisms between E2F1 and CIP2A are essential for activation of senescence and response to chemotherapy, providing further evidence for the combined roles of the p53- and the pRb functional pathways in this respect.^26^

As a response to DNA damage and stress, both apoptosis and cellular senescence can be activated in parallel, but also separately. Why some cells respond with apoptosis, while other respond with senescence upon the same treatment is still not fully understood. It has previously been reported that treatment of a colorectal cancer cell line with low doses of doxorubicin induces senescence rather than apoptosis,^27^ but it has also been suggested that in cells that are unable to induce apoptosis, senescence may act as a ‘backup’ response, strongly contributing to the treatment outcome.^28^ Furthermore, our results are supported by several *in vivo* studies, indicating that upon chemotherapy treatment, the role of p53 as a tumor suppressor may be mediated through induction of senescence rather than apoptosis.^29,30^

The MCF-7 cells may be regarded as the most suitable model in our experiments since they are wild type for both *TP53* and *RB1*. One may assume that cell lines with mutations in *TP53* and or *RB1* would adapt to the situation and activate compensatory signaling pathways, making them independent of *TP53*/*RB1* function. However, in our experiments, we found the results in HTB-122 cells to resemble the results form MCF-7, even if HTB-122 harbors a mutated *TP53* (R249S): Upon chemotherapy treatment, similar changes were observed in cell cycle distribution and, further, when assessing activation of apoptosis in HTB-122 cells, the effects of *TP53* and/or *RB1* inactivation were limited in this cell line as well. This may indicate that some of the functions of *TP53* R249S could influence the cells response to cytotoxic stress.

Taken together, the results from these two cell lines support our previous data from breast cancer patients *in vivo*, strengthening the proposed model which states that resistance to chemotherapy might depend on a ‘two-pathway-hit’, involving inactivation on both the p53- and the Rb-pathway.^22^ Notably, this may also influence resistance to chemotherapy in other tumor types: Zhu et al recently suggested, based on studies of primary and metastatic prostate cancer, that genetic co-inactivation of both pRb and p53 could explain why more advanced prostate cancers become chemotherapy resistant as the tumors evolve.^25,31,32^

Although p53 and pRb are considered to be two of the most important proteins responsible for induction of apoptosis and cell cycle arrest, it is important to note that the involvement and functionality of other alternative pathways could also be involved in the response to chemotherapy in a setting where both p53 and pRb are inactivated. Such alternative pathways could be the cause for why our results from MCF-7 and HTB-122 were not properly mirrored in T47D and CRL2324. Regarding T47D and CRL2324 cells, the overall percentage of apoptotic cells were very low in our experiments. However, it should be noted that, despite the apparent resistance to the applied doxorubicin dose, T47D cells did actually reveal some changes in cell cycle distribution, resembling the results in MCF-7 cells. Alternative pathways, independent of p53, such as ATM-Chk2-cdc25 signaling, has been found to influence the cellular responses to genotoxic stress,^33^ and it may be that such a signaling route is active in this cell line.

To conclude, the present study shows that concomitant inactivation of the p53- and the Rb-pathway after treatment with a DNA damaging chemotherapeutic drug, leads to altered cell cycle, minor changes in apoptosis and a decrease of cells that are entering a senescent state, compared to inactivation of the two pathways individually. Additionally, our findings indicate these effects to be independent of the cells ER- and PgR-status.

## Materials and methods

### Cell lines and cultures

The breast cancer cell lines used in the present study are listed in Table 1. The cell lines were preferred based on their ER, PgR, Her-2 characteristic, in addition to their *TP53* and *RB1* mutational status. Except for MCF-7, being WT for *TP53* as well as *RB1*, the other cell lines (T47D, HTB-122 and CRL2324) each harboring a *TP53* mutation previously characterized as a ‘gain of function’ (GOF) mutation and with loss of the normal allele. HTB-122 in addition harbor partly deleted *RB1,* possibly causing a non-functional pRb protein.^34–37^

Before use, the cell lines identities were confirmed by STR based DNA fingerprinting using the AmpF*l*STR Profiler Plus and Cofiler kits (Applied biosystems by Life Technologies, Carlsbad, CA, USA) according to the manufacturer’s instructions. All cell lines and medium are purchased from ATCC, and all of the different medium were supplemented with 10% FBS (Lonza, Basel, Switzerland), 1% l-Glatamine (Lonza) and 1% Penicillin Streptavidin (Lonza), and in addition McCoys were supplemented with 1 *μ*l/ml insulin (Sigma, St Louis, MO, USA) for the T47D cells. In all the assays the cells were transfected with siRNA for 24 h, followed by treatment with dimethyl sulfoxide (DMSO, referred to as untreated cells, as negative control, Sigma) or 1 *μ*M doxorubicin (Nycomed, Zurich, Switzerland) for 24 h, with the exception of the senescence assay (see below).

### siRNA treatment

Cells were treated with ON Target siRNA smart pool (Darmacon, Lafayette, CO, USA) against *TP53* and/or *RB1* or control siRNA. Transfection was performed using Lipofectamin RNAiMAX (Invitrogen, Carlsbad, CA, USA) according to manufacturer’s instructions and monitored by Western blot analysis. We applied siRNA-mediated knockdown of *TP53* and *RB1* in all the cell lines, for more uniform comparison.

### Doxorubicin treatment

Cells were treated with 1 *μ*M doxorubicin for assessment of cell cycle phase distribution and induction of apoptosis. This fixed dose was applied across all cell lines in order to mimic the effective doses of doxubicin used in patients. For the assessment of senescence, the long incubation time made this dose non-feasible and a reduced dose of 0.5 *μ*M doxorubicin was applied for this assay (see details for the assays below).

### Western blot analysis

The cells were harvested with Trypsin EDTA (Lonza) and lysed with IPH buffer containing protease and phosphatase inhibitor (Roche, Basel, Switzerland). Further, the cells were washed with 1xPBS and loaded on to 12% polyacrylamide gels (BioRad, Hercules, CA, USA) with PS11 protein ladder (GeneOn, Ludwigshafen, Germany) and blotted for 7 min with Trans-blot Turbo system (BioRad) on to 0.2 *μ*M nitrocellulose membranes (BioRad). Membranes were blocked with 5% non-fat milk in TBS-Tween_0.05%_, and visualized by anti-p53 (Sigma), anti-pRb (Cell Signaling, Danvers, MA, USA) and HRP-tagged secondary antibody (Sigma) in 1% dry milk in TBS-Tween_0.05%_ on Fuji-LAS 4000 (GE Healthcare Life Science, Little Chalfont, UK). As loading control, anti-Actin (Thermo Scientific, Carlsbad, CA, USA) was used as primary antibody.

### Cell cycle progression analysis

The siRNA transfected cells, treated with DMSO or 1 *μ*M doxorubicin were incubated 5 min at 37 °C in lysis buffer containing Hoechst (Chemometec, Allerød, Denmark). Cells were further added stabilizing buffer (Chemometec) before analyzing with the NucleoCounter 3000 for cell cycle status by DNA quantification. The analysis was repeated in three independent experiments with three independent transfections.

### Apoptosis analysis by Annexin V staining

siRNA transfected cells were treated with either DMSO or 1 μM doxorubicin before harvesting with Trypsin EDTA (Lonza). The cells were then washed in 1xPBS, incubated for 15 min at 37 °C in Annexin V buffer with Annexin V (Biotium) and Hoechst (Chemometec). Next, the cells were washed once in Annexin V buffer (Biotium, Fremont, CA, USA), before they were re-suspended in Annexin V buffer with 4% PI (Chemometec). Apoptosis assay was performed on a NucleoCounter 3000 instrument (Chemometec) with identification of apoptotic cells. The assay was repeated in triplicate with three independent transfections. In order to avoid major overlap with signals from PI (535/617 nm), or from potential remains of doxorubicin molecules (480/~570 nm) we applied the CF488A Annexin V conjugate (490/515 nm; Chemometec) and a custom filter setting on the NucleoCounter instrument.

### Senescence assay: *β*-galactosidase activity staining

Transfected MCF-7 cells were treated with DMSO or 0.5 *μ*M doxorubicin for 7 days before staining for *β*-galactosidase activity with the Senescence *β*-galactosidase Staining Kit (Cell Signaling), according to the manufacturer’s recommendations. Staining of the cells was performed by incubation of the cells in staining solution for 16 h, in a cultivation incubator with humidified atmosphere, without CO_2_ at 37 °C. The senescent cells were observed as blue *β*-galactosidase positive cells upon microscopy (Nikon eclipse TS100) and quantitated by counting performed by two independent investigators, blinded to sample identity and each other’s results. The experiment was repeated in triplicates and in each experiment a minimum of 164 cells was counted for each sample treated with doxorubicin and 383 cells for the untreated sample.

### Statistics

Potential differences in cell cycle distribution and apoptosis, as well as for the interaction between the induction of senescence and activation of apoptosis, were assessed by univariate analysis of variance (assessing the cell cycle distribution, all phases were included in the univariate analysis of variance). All *P*-values are reported as two-sided.

## Figures and Tables

**Table 1:** Breast cancer cell lines characterizations

*Cell line*	*ATCC no.*	*Medium*	*Subtype*	*Tumor type*	*ER*	*PgR*	*Her-2*	TP53	RB1
MCF-7	HTB-22	EMEM	L	Met AC	+	+	−	WT	WT
T47D	HTB-133	McCoys	L	IDC	+ (low)	+ (high)	−	GOF Mutation (L194F) Homozygous	WT
HTB-122	BT-549	RPMI	B	IDC	−	−	−	GOF Mutation (R249S) Homozygous	Frameshift (c.265_607-del343) Homozygous
CRL2324	HCC1395	RPMI	B	DC	−	−	−	GOF Mutation (R175H) Homozygous	WT

Abbreviations: B, Basal B subtype; L, luminal subtype; DC, ductal carcinoma; IDC, invasive ductal carcinoma; Met AC, metastatic adenocarcinoma.
